# Rapid Molecular Tests for Detecting Respiratory Pathogens Reduced the Use of Antibiotics in Children

**DOI:** 10.3390/antibiotics10030283

**Published:** 2021-03-10

**Authors:** Yu Kyung Kim, Jong Ho Lee, Sae Yoon Kim, Ji Young Ahn, Kwang Hae Choi, Young Hwan Lee, Kyung Mi Jang, Yong Sauk Hau, Jae Min Lee

**Affiliations:** 1 Department of Clinical Pathology, School of Medicine, Kyungpook National University, Daegu 41944, Korea; kimyg@knu.ac.kr; 2 Department of Laboratory Medicine, Yeungnam University College of Medicine, Daegu 42415, Korea; leejongho@ynu.ac.kr; 3 Department of Pediatrics, College of Medicine, Yeungnam University, Daegu 42415, Korea; sysnow88@hanmail.net (S.Y.K.); jy4413@gmail.com (J.Y.A.); choi8819@gmail.com (K.H.C.); yhlee@med.yu.ac.kr (Y.H.L.); fortune001j@gmail.com (K.M.J.); 4 Department of Business Administration, School of Business, Yeungnam University, Gyeongsan 38541, Korea; augustine@yu.ac.kr

**Keywords:** acute respiratory infection, antimicrobials, multiplex polymerase chain reaction, respiratory pathogen, FilmArray Respiratory Panel

## Abstract

Multiplex polymerase chain reaction (mPCR) is increasingly being used to diagnose infections caused by respiratory pathogens in pediatric inpatient facilities. mPCR assays detect a broader array of viruses, with higher specificity and sensitivity and faster turnaround than previous assays. We adapted the FilmArray Respiratory Panel (FA-RP) for diagnosing respiratory infections. FA-RP is an in vitro mPCR assay that simultaneously and rapidly (in about 1 h) detects 20 pathogens directly from respiratory specimens. Here, we studied the clinical efficacy of FA-RP in children who underwent testing for respiratory pathogens at Yeungnam University Hospital from November 2015 to August 2018. From November 2015 to June 2016, routine mPCR testing was performed on nasopharyngeal swabs using the routine mPCR kit. From November 2016 to July 2018, mPCR testing was performed using FA-RP. A total of 321 tests by routine mPCR and 594 tests by FA-RP were included. The positive detection rates for routine mPCR and FA-RP were 71.3% and 83.3%, respectively. FA-RP reduced the lead time, waiting time, turnaround time, intravenous (IV) antibiotic use, and length of hospital stay for pediatric patients. The decreased use of antibiotics is expected to reduce antibiotic resistance in children.

## 1. Introduction

Acute respiratory infection (ARI) is a leading cause of hospitalization for young children. Among infants in the United States, the median cost of hospitalization due to infectious disease was 2235 USD, with total annual hospital costs of approximately 690 million USD [1]. Respiratory viruses are the most infectious pathogens in humans, with worldwide distribution and broad diversity in type, antigenicity, and patterns of infections; this makes understanding their infection patterns difficult [2]. ARI is the most common illness, regardless of age or sex [3]. The burden of disease caused by ARIs is substantial, and ARIs are the third most common cause of death worldwide [4]. Infants and young children are particularly vulnerable to respiratory disease. Among them, pneumonia is the predominant cause of childhood mortality, and it causes nearly 1.3 million deaths per year. Early childhood respiratory infection or environmental exposures may lead to chronic disease in adulthood [5,6].

Although bacteria were previously considered to be the principal etiological agents of severe respiratory tract infections, the global impact of respiratory viruses has been increasingly recognized in recent years [7,8,9,10]. Between 2001 and 2010, there were estimated to be 43 million emergency department (ED) visits for patients less than 5 years old that resulted in diagnoses of ARI (354 per 1000 ED visits) [11]. There were 126 million ED visits with diagnoses of ARI, and antibiotics were prescribed in 61% of the cases in these periods. Significant progress has been made toward the reduction of antibiotic use in pediatric patients with ARI. Therefore, between 2001 and 2010, antibiotic use decreased for patients aged <5 years presenting with antibiotic-inappropriate ARI.

The emergence of antibiotic-resistant bacteria is a serious global challenge. Antibiotic resistance develops when bacteria adapt and grow in the presence of antibiotics. As the development of resistance is linked to the frequency of antibiotic use, the misuse and overuse of antibiotics hastens the development of bacterial drug resistance, rendering existing antibiotics less effective. In Korea, antimicrobials were most frequently prescribed to children younger than 10 years of age. The Korean government has implemented a series of healthcare policies that have resulted in the decrease of antibiotic prescription for the treatment of upper respiratory infections because the causative agents were mostly viral [12].

Multiplex polymerase chain reaction (mPCR) for the diagnosis of respiratory pathogens is increasingly being used in pediatric inpatient facilities [13,14]. The use of mPCR testing for respiratory viruses among hospitalized patients has been significantly associated with decreased healthcare resource utilization, including decreased use of antibiotics and chest radiography and increased use of isolation precautions [15]. The availability of the test results of mPCR for respiratory pathogens at a clinically actionable time can influence antibiotic prescription; in other words, antibiotic use might be reduced if the results of virus checks are obtained rapidly. The FilmArray Respiratory Panel (FA-RP) (BioFire Diagnostics, Inc., Salt Lake City, UT, USA) is the first FDA-cleared assay for the qualitative detection of nucleic acid targets from 20 respiratory pathogens with a turnaround time (TAT) of 1 h [16]. The sensitivity and specificity of this rapid test were similar to the existing diagnostic test. The workflow of FA-RP was simple, making it suitable for introducing emergency testing. We have previously reported that the use of the FA-RP increases diagnostic efficiency and reduces turnaround time (TAT) in laboratories [17].

The aim of this study was to determine the real-world clinical impact of FA-RP results on antibiotic use and hospital stay, particularly if antibiotic use was reduced as a result of decreased lead time.

## 2. Results

### 2.1. Baseline Characteristics of Patients

**Figure 1:**
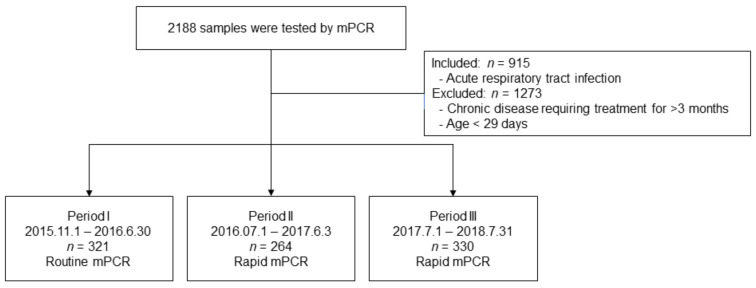
Patient enrollment flowchart.

Figure 1 shows the flowchart of patient enrollment. The number of patients included in the study in periods I, II, and III were 321, 264, and 330, respectively. The age or sex distribution of patients between periods was not significantly different. In laboratory findings, white blood cell count, hemoglobin levels, aspartate aminotransferase levels, and alanine aminotransferase levels were not significantly different between periods. The platelet count was elevated during period III but was still within the normal range (322,000 ± 124,000 vs. 358,000 ± 128,000; *p* = 0.007). C-reactive protein levels were not significantly different between periods, and lactate dehydrogenase levels were elevated during period III. However, these test results were not clinically significant (Table 1). The percentages of patients with upper respiratory tract infection (URI), lower respiratory tract infection (LRI), and both URI and LRI (URI + LRI) were 28.3%, 64.4%, and 7.2%, respectively.

### 2.2. Virus Detection

Positive detection rates between the routine test and FA-RP were significantly different (71.3% vs. 83.3%; *p* < 0.0001, respectively). For routine tests performed during period I, negative results were obtained in 28.3% of samples, while 56.7% of samples tested positive for one virus and 15.0% tested positive for two viruses. However, in FA-RP, viruses were detected in 83.3% of samples. One virus was detected in 33.8% of all tests; two viruses were detected in 27.9% of samples; and more than three viruses were detected in 20.2% of samples. Bacteria were detected in 3.5% of samples and virus–bacteria co-infection was seen in 13 (2.2%) of samples tested by FA-RP (Table 2).

**Figure 2:**
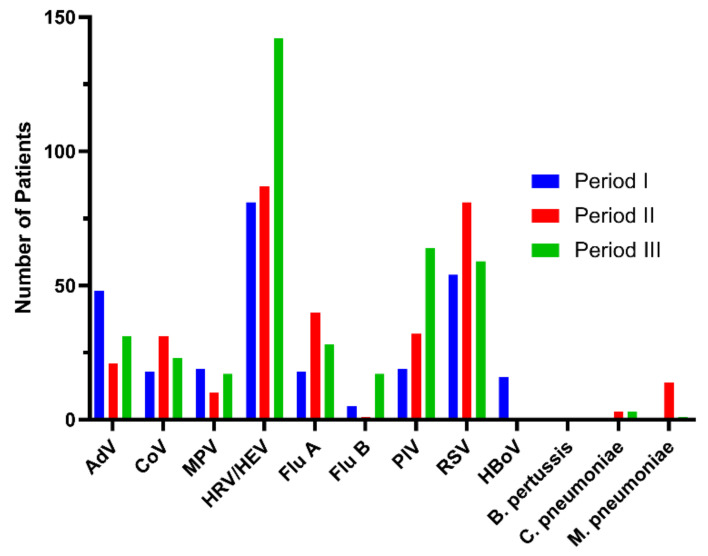
The results of positive respiratory virus tests according to period.

Human rhinovirus (HRV) was the virus most frequently detected during period I (n = 72, 21.6%), followed by respiratory syncytial virus (RSV) (n = 53, 15.9%) and adenovirus (AdV) (n = 48, 14.4%). During period III, HRV and human enterovirus (HEV) were most frequently detected (n = 156, 42.0%), followed by RSV (59, 16%) and parainfluenza viruses (PIV) (n = 50, 13%) (Table 3, Figure 2).

### 2.3. Test Time, Antibiotic Use, and Hospital Stay

**Figure 3:**
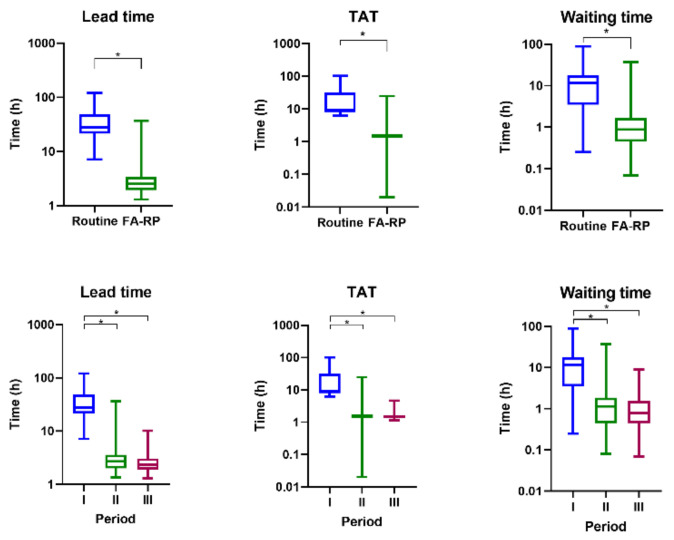
Comparison of test time according to the multiplex polymerase chain reaction (mPCR) test and period; * means two values were statistically significant (*p* < 0.05).

Lead time, waiting time, and TAT were shorter in the FA-RP group (*p* < 0.001) (Figure 3).

**Figure 4:**
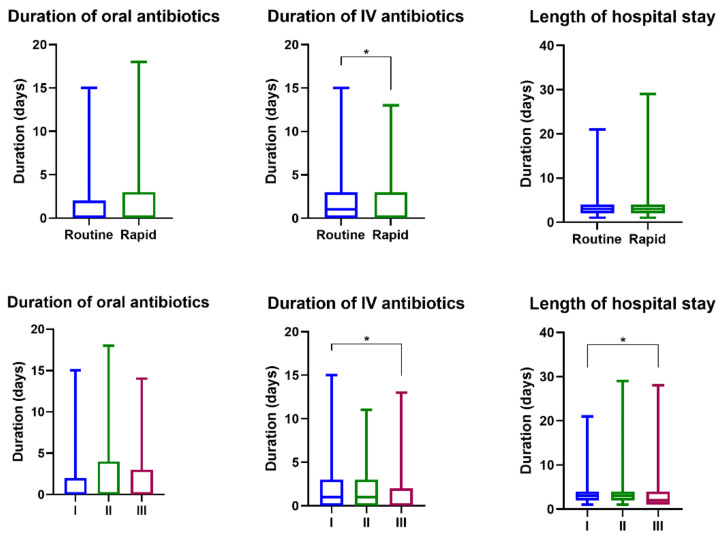
Comparison of antibiotic use and hospital stay according to the mPCR test and period; * means two values were statistically significant (*p* < 0.05).

The frequency of intravenous (IV) antibiotic use during period I using the routine test was 51.7%, and the rates of antibiotic use during periods II and III using FA-RP were 52.7% and 39.4%, respectively. In particular, the frequency of IV antibiotic use decreased significantly during period III (*p* = 0.002). There was no significant difference in the frequency of oral antibiotic use during the study period. There was no significant difference between the frequency and duration of IV antibiotic use between period I and period II. However, between period I and period III, the frequency of use of IV antibiotics, duration of use, and period of use of IV + oral antibiotics decreased significantly (Table 4, Figure 4). The duration of IV antibiotic use was significantly reduced in the FA-RP group when compared to the routine test group (*p* = 0.015). The length of hospital stay was significantly reduced in period III when compared to period I (*p* = 0.004).

Sixty-nine patients were discharged without hospitalization after mPCR tests in the ED. Of these, 59 patients underwent FA-RP testing, 49 (83%) of whom tested positive and 10 (16.9%) tested negative. Ten discharged patients underwent routine testing, six of whom tested positive and four of whom tested negative.

## 3. Discussion

We compared two respiratory pathogen tests performed during a three-year period and compared the clinical management and patient characteristics with respect to the method of pathogen testing. We have reported in a previous study that the use of FA-RP significantly reduces the mean waiting time, TAT, and lead time compared to the routine test [17]. While a routine mPCR method that can simultaneously perform nucleic acid amplification and extraction is commonly used, the routine mPCR test cannot be used as an emergency test as it takes a long time to yield results. As the routine mPCR test involved batch operation, reviews of the results and follow-up actions were performed only after initial isolation or treatment. Even if the sample was delivered quickly, the test result was not revealed quickly, and hence, waiting time, sample delivery, and sample reception time were delayed. Previous studies on the usefulness of FA-RP as a detector of respiratory viruses found that the test was effective in detecting viruses that went undetected by previous routine mPCR methods. This was of interest as it indicated the usefulness of the technique in reducing both unnecessary antibiotic use and invasive investigation. However, the role mPCR can play in the diagnosis of ARI is unclear because the use of mPCR has not yet been recommended in national and international guidelines due to a lack of research on the cost effectiveness [18].

The impact of reverse transcription (RT)-PCR testing on clinical management, antibiotic use, and the length of hospital stay for children with respiratory infections has been studied, with respiratory sample results being reported to clinicians within 12–36 h for the study group and after 4 weeks in the control group. In this case, rapid reporting did not result in a change in patient care. There were no significant differences between the groups with respect to hospital admission, length of hospital stay, or duration of antibiotic use. The authors concluded that although RT-PCR testing had a high yield of viral diagnoses, rapid communication of these results did not lead to shorter hospital stay, decreases in hospital admissions, or decreased antibiotic use for children with ARIs [14].

In a similar randomized trial in adults, the mPCR results were available in 24 h for the study group and in 7 days for the control group. mPCR testing for respiratory viruses with results available within 24 h did not reduce the consumption of bacterial antibiotics or the length of hospital stay in adults in the emergency unit [19]. However, Brendish et al. reported that a point-of-care test with a TAT of ≤1.6 h was associated with earlier hospital discharge and earlier discontinuation of antibiotics compared to tests with longer TATs [20]. In our study, the TAT was less than 3 h, shorter than that in the previous studies. Once the mPCR results were obtained, the physician could apply them to clinical practice. This led to a decrease in the frequency and duration of antibiotic use. Thus, early notification of the test results led to significant changes in clinical practice. Although there is a difference in the frequency of the detection of viruses and bacteria according to the period, the difference between the period of antibiotic use and the frequency of use of antibiotics due to this is not likely to be significant because the incidence of *pertussis* and *Mycoplasma* and *Chlamydia* infections during periods II and III was not high.

In a previous randomized controlled trial on the impact of early and rapid diagnosis of viral infections in children with febrile respiratory tract illness in the ED, children were randomly assigned to either undergo mPCR or routine care [21]. mPCR testing was performed by the research nurses at triage and hand-delivered to the laboratory. The results were added to the patient chart as soon as they became available, and as such, this trial intervention did not lend itself to blinding. Patient treatment was otherwise comparable for both the groups. For the control patients, the mPCR tests could be ordered by the physician after assessment and performed at the bedside by the usual bedside nurses. The TAT of the mPCR tests was 30–150 min. In their study, no statistically significant differences were found between the ED visit length, rate of ancillary testing, or antibiotic prescription rate during the ED visit between the study groups. There was, however, a significant reduction in antibiotic prescription after ED discharge. As in our study, the short TAT seems to have resulted in the reduction of antibiotic use.

The Infectious Disease Society of America guidelines suggest the use of rapid viral testing for respiratory pathogens as a way to reduce the inappropriate use of antibiotics. However, as of now, it is only a weak recommendation due to low-quality evidence. Although rapid viral testing has the potential to reduce the inappropriate use of antibiotics, the results have been inconsistent [22]. Our results suggest that mPCR testing with a TAT of no more than 3 h is important for reducing antibiotic use as a step toward antibiotic stewardship.

Kreitmeyr et al. conducted a prospective study on antibiotic stewardship programs (ASPs) that aim to reduce antibiotic consumption. ASPs targeted the infectious diseases (ID) ward rounds (prospective audit with feedback), ID consultation services, and internal guidelines on empiric antibiotic therapy. The study concluded that the implementation of an ASP was associated with a profound improvement in rational antibiotic use, and no adverse effects regarding the length of hospital stay or in-hospital mortality were observed [23].

In our study, with the introduction of the FA-RP test, the test time was shortened, but there was no significant difference in the frequency and duration of antibiotic use between periods I and II. However, over time, in period III, the frequency and duration of antibiotic use were significantly decreased. This suggests that despite the introduction of effective tests, it takes time to implement ASPs and to change clinical practice.

In the study of young Italian doctors’ knowledge, attitudes, and practice of ASP, only 20–40% of doctors answered vancomycin-resistant enterococci (VRE), carbapenem-resistant Enterobacteriaceae (CRE), extended-spectrum β-lactamase-producing enterobacteria (ESBL), and methicillin-resistant *Staphylococcus aureus* (MRSA) correctly. In addition, 81% of participants said that ASP was not properly handled during their medical training, and 71% said they did not learn appropriate examples from their tutors. Therefore, proper ASP education for medical doctors is very desperately needed [24].

In this study, we analyzed the usefulness of two viral tests performed at different times in patients who visited the hospital for respiratory disease and fever. Since the emphasis was placed on comparing the effectiveness of the tests, the patients’ clinical characteristics were not analyzed in detail.

In our study, there were viral–viral co-infections and viral–bacterial co-infections. Several studies have been conducted on the clinical significance of viral co-infection. It is known that co-infection between viruses and interference between viruses can occur. In particular, it is known that RSV accounts for most childhood infections [25]. Although this is beyond the scope of the present study, as various co-infections are confirmed due to the activation of mPCR tests, further studies on this aspect are warranted.

While complex policy changes and management are an important part of ASPs, it is a challenging task, requiring time, effort, and careful implementation. Our findings are significant as they demonstrate that simply changing to an mPCR kit with a faster TAT resulted in reduced antibiotic use. Careful management of antibiotics, the use of ASPs, and targeted medical education to pediatricians and pediatric hospitals are important for the overall reduction of antibiotic use [22,23,26]. By providing evidence for an efficient test for pediatric ARIs, we have improved the ability of clinicians to implement good ASPs.

In conclusion, despite the limitations of the study, TAT was shortened through mPCR tests, and it was confirmed that the use of antibiotics could be reduced. In the future, it will be necessary to apply a rapid mPCR test to pediatric patients who need to consider antibiotic prescription due to febrile illness and respiratory disease in order to reduce duration of antibiotics, hospitalization period, and medical costs.

## 4. Materials and Methods

We conducted a retrospective cohort study of children hospitalized at Yeungnam University Hospital from November 2015 to August 2018, who underwent testing for respiratory pathogens either in the ED prior to admission or within the first 2 days of hospitalization. Virus tests were performed on patients with fever accompanied by diseases of the respiratory system, such as URI, bronchiolitis, pneumonia, croup, and tonsillitis. We excluded patients with chronic disease requiring treatment for more than 3 months and infants under 29 days of age. From November 2015 to June 2016 (period I), routine mPCR testing was performed on nasopharyngeal swabs by using the AnyplexTM II RV16 detection kit (RV16; Seegene, Seoul, Korea). From July 2016 to July 2018 (periods II and III), newly adopted mPCR testing was performed on nasopharyngeal swabs by using the FA-RP (BioFire Diagnostics, Inc., Salt Lake City, UT, USA). We divided this period into periods II (2016.7.1 to 2017.6.30) and III (2017.7.1 to 2018.7.31). After the introduction of the rapid mPCR test method, we conducted a study by dividing the period by one year to check whether medical practices such as antibiotic prescription and hospital stay changed.

The medical records of enrolled patients were retrospectively reviewed to confirm laboratory results, identify patient characteristics, and determine the duration of oral and intravenous (IV) antibiotic use and that of hospitalization. mPCR testing was used to detect viruses and bacteria. Positive detection rates of viruses and bacteria were determined. The waiting time (time from prescription to submission of a specimen to the laboratory), TAT (time from submission of a specimen to the final result), and lead time (time from prescription to the final result) of the routine test and FA-RP test were analyzed. The indications for antibiotic use were a negative result on the mPCR test; unstable clinical symptoms before the virus test result; or elevated levels of inflammatory markers such as erythrocyte sedimentation rate (ESR), C-reactive protein (CRP), and procalcitonin.

This study was approved by the institutional review boards (IRB) of Yeungnam University Hospital (IRB approval number: YUMC 2019-11-026). Informed consent was waived due to the retrospective nature of this study.

### 4.1. Routine Test

The routine mPCR test for detecting respiratory viruses was performed using the Anyplex II RV16 detection kit. RV16 can detect 16 viruses: AdV, influenza A, influenza B, PIV 1–4, HRV, bocaviruses, coronavirus (229E, NL63, OC43), HEV, RSVA, RSVB, and metapneumovirus (MPV) [27]. The routine test is run in batch operation and is performed 3–4 times a week.

### 4.2. FA-RP

The newly adopted mPCR test for detecting respiratory viruses was performed using the FA-RP, which targets 20 viruses and bacteria: AdV, coronavirus (HKU1, NL63, 229E, and OC43), MPV, HRV/HEV, influenza A, influenza A/H1, influenza A/H3, influenza A/H1-2009, influenza B, PIV 1–4, RSV, *Bordetella pertussis*, *Chlamydia pneumoniae*, and *Mycoplasma pneumoniae* [16]. The FA-RP test is a point-of-care test, with a run time of 24 h (any time of the day).

### 4.3. Statistical Analyses

The statistical package SPSS version 25.0 was used to analyze the data (SPSS Inc., Chicago, IL, USA). Categorical variables were compared using Pearson’s chi-square test to compare patient characteristics, frequency of pathogen, and antibiotic use. The *t*-test was used to compare laboratory values, duration of hospital stay, and antibiotic use. Statistical significance was defined at *p* < 0.05.

## Figures and Tables

**Table 1:** Baseline characteristics of patients.

	Routine Test	FA-RP			*p*-Value
Period	I	II–III	II	III	
	2015.11.1–2016.6.30	2016.7.1–2018.7.31	2016.7.1–2017.6.30	2017.7.1–2018.7.31	
N	321	594	264	330	
Sex (Male %)	179 (55.8)	360 (60.6)	158 (59.8)	202 (61.2)	0.344
Age (years)	3.5 ± 4.2	3.4 ± 4.2	3.3 ± 4.4	3.5 ± 4.1	0.454
WBC (/µL)	11,099 ± 5157	11,689 ± 5093	11,746 ± 5026	11,655 ± 5140	0.126
Hb (g/dL)	12.0 ± 1.1	12.2 ± 1.2	12.2 ± 1.2	12.2 ± 1.2	0.069
Platelets (/µL)	322,000 ± 124,000	358,000 ± 128,000	368,000 ± 116,000	353,000 ± 134,000	0.007
CRP (mg/dL)	2.2 ± 3.1	1.6 ± 2.2	2.0 ± 2.5	1.4 ± 2.0	0.051
AST (IU/L)	42 ± 34	44 ± 62	47 ± 95	42 ± 27	0.963
ALT (IU/L)	27 ± 48	31 ± 98	37 ± 151	27 ± 43	0.275
LDH (IU/L)	542 ± 134	582 ± 200	539 ± 160	602 ± 214	0.009

*p*-values were calculated between periods I (2015.11.1–2016.6.30) and II–III (2016.7.1–2018.7.31).

**Table 2:** Comparison of virus detection between periods.

	Routine Test	FA-RP			*p*-Value
Period	I	II–III	II	III	
	2015.11.1–2016.6.30	2016.7.1–2018.7.31	2016.7.1–2017.6.30	2017.7.1–2018.7.31	
N	321	594	264	330	
Detection, n (%)	230 (71.7)	495 (83.3)	217 (82.2)	278 (84.2)	<0.001
Negative, n (%)	91 (28.3)	99 (16.7)	47 (17.8)	52 (15.8)	
Virus detection, n (%)	230 (71.7)	487 (82.0)	211 (79.9)	276 (83.6)	<0.001
1 virus	182 (56.7)	201 (33.8)	95 (36.0)	106 (32.1)	
2 viruses	48 (15.0)	166 (27.9)	62 (23.5)	104 (31.5)	
≥3 viruses	0	120 (20.2)	54 (20.5)	66 (19.9)	
Bacterium detection, n (%)	0	21 (3.5)	17 (6.4)	4 (1.2)	
Viral–bacterial co-infection, n (%)	0	13 (2.2)	11 (4.2)	2 (0.6)	

*p*-values were calculated between periods I and II–III.

**Table 3:** Pathogens causing respiratory infections detected in different periods.

	I	II	III
Number of patients, n (%)	321 (100)	264 (100)	330 (100)
AdV	48 (15.0)	21 (8.0)	31 (9.4)
CoV	15 (5.6)	31 (11.7)	23 (7.0)
MPV	19 (5.9)	10 (3.8)	17 (5.2)
HRV/HEV	81 (25.2)	87 (33.0)	142 (43.0)
Flu A	18 (5.6)	40 (15.2)	28 (8.5)
Flu B	5 (1.6)	1 (0.4)	17 (5.2)
PIV	19 (5.9)	32 (12.1)	64 (19.4)
RSV	54 (16.8)	81 (30.7)	59 (17.9)
HBoV	16 (5.0)	0 (0)	0 (0)
*Bordetella pertussis*	0	0 (0)	0 (0)
*Chlamydophila pneumoniae*	0	3 (1.1)	3 (0.9)
*Mycoplasma pneumoniae*	0	14 (5.3)	1 (0.3)

AdV, adenovirus; CoV, coronavirus; MPV, metapneumovirus; HRV, human rhinoviruses; HEV, human enterovirus; Flu, influenza; PIV, parainfluenza virus; RSV, respiratory syncytial virus; HBoV, bocaviruses.

**Table 4:** Comparison of antibiotic use and hospital stay according to mPCR test and period.

	Routine Test	FA-RP					
Period	I	II–III	II	III	*p*-Value ^a^	*p*-Value ^b^	*p*-Value ^c^
	2015.11.1–2016.6.30	2016.7.1–2018.7.31	2016.7.1–2017.6.30	2017.7.1–2018.7.31			
N	321	594	264	330			
Waiting time (hours)	14.2 ± 13.7	1.4 ± 2.0	1.6 ± 2.7	1.1 ± 1.0	<0.001	<0.001	<0.001
TAT (hours)	21.7 ± 18.7	1.6 ± 1.1	1.7 ± 1.5	1.6 ± 0.4	<0.001	<0.001	<0.001
Lead time (hours)	35.9 ± 20.4	3.0 ± 2.2	3.3 ± 3.0	2.7 ± 1.1	<0.001	<0.001	<0.001
Frequency of use of IV antibiotics (%)	51.7	45.3	52.7	39.4	0.821	0.002	0.063
Frequency of use of oral antibiotics (%)	26.5	31.0	35.2	27.6	0.022	0.753	0.154
Duration of oral antibiotic use (days)	1.7 ± 3.2	1.7 ± 3.0	2.1 ± 3.4	1.4 ± 2.7	0.122	0.952	0.285
Duration of IV antibiotic use (days)	1.7 ± 2.2	1.4 ± 2.0	1.7 ± 2.1	1.2 ± 2.0	0.619	<0.001	0.015
Duration of IV + oral antibiotic use (days)	3.4 ± 4.3	3.1 ± 4.2	3.8 ± 4.5	2.7 ± 3.9	0.359	0.019	0.364
Hospital LOS (days)	3.2 ± 2.2	3.2 ± 2.7	3.5 ± 2.8	3.0 ± 2.6	0.189	0.004	0.188

TAT, turnaround time; LOS, length of stay. ^a^: *p*-values were calculated between periods I and II. ^b^: *p*-values were calculated between periods I and III. ^c^: *p*-values were calculated between periods I and II–III.

## Data Availability

The data presented in this study are available on request from the corresponding author.

## References

[1] Yorita K.L., Holman R.C., Sejvar J.J., Steiner C.A., Schonberger L.B. (2008). Infectious disease hospitalizations among infants in the United States. Pediatrics.

[2] Mahony J.B. (2008). Detection of respiratory viruses by molecular methods. Clin. Microbiol. Rev..

[3] Monto A.S. (2002). Epidemiology of viral respiratory infections. Am. J. Med..

[4] GBD Mortality and Causes of Death Collaborators (2015). Global, regional, and national age-sex specific all-cause and cause-specific mortality for 240 causes of death, 1990-2013: A systematic analysis for the Global Burden of Disease Study 2013. Lancet.

[5] Zar H.J., Ferkol T.W. (2014). The Global Burden of Respiratory Disease-Impact on Child Health. Pediatr. Pulm..

[6] Wisnivesky J., De-Torres J.P. (2019). The Global Burden of Pulmonary Diseases: Most Prevalent Problems and Opportunities for Improvement. Ann. Glob. Health.

[7] George M., Ahmad S.Q., Wadowski S. (2015). Community-acquired pneumonia among U.S. children. N. Engl. J. Med..

[8] Weinberger M. (2015). Community-acquired pneumonia among U.S. children. N. Engl. J. Med..

[9] Jain S., Self W.H., Wunderink R.G., Fakhran S., Balk R., Bramley A.M., Reed C., Grijalva C.G., Anderson E.J., Courtney M. (2015). Community-acquired pneumonia requiring hospitalization among U.S. adults. N. Engl. J. Med..

[10] Jain S., Williams D.J., Arnold S.R., Ampofo K., Bramley A.M., Reed C., Stockmann C., Anderson E.J., Grijalva C.G., Self W.H. (2015). Community-acquired pneumonia requiring hospitalization among U.S. children. N. Engl. J. Med..

[11] Donnelly J.P., Baddley J.W., Wang H.E. (2014). Antibiotic utilization for acute respiratory tract infections in U.S. emergency departments. Antimicrob. Agents Chemother..

[12] Kim Y.A., Park Y.S., Youk T., Lee H., Lee K. (2018). Changes in antimicrobial usage patterns in Korea: 12-Year analysis based on database of the national health insurance service-national sample cohort. Sci. Rep..

[13] Wishaupt J.O., Versteegh F.G., Hartwig N.G. (2015). PCR testing for paediatric acute respiratory tract infections. Paediatr. Respir. Rev..

[14] Wishaupt J.O., Russcher A., Smeets L.C., Versteegh F.G., Hartwig N.G. (2011). Clinical impact of RT-PCR for pediatric acute respiratory infections: A controlled clinical trial. Pediatrics.

[15] Subramony A., Zachariah P., Krones A., Whittier S., Saiman L. (2016). Impact of multiplex polymerase chain reaction testing for respiratory pathogens on healthcare resource utilization for pediatric inpatients. J. Pediatrics.

[16] Babady N.E. (2013). The FilmArray® respiratory panel: An automated, broadly multiplexed molecular test for the rapid and accurate detection of respiratory pathogens. Expert Rev. Mol. Diagn..

[17] Lee J.M., Lee J.H., Kim Y.K. (2018). Laboratory impact of rapid molecular tests used for the detection of respiratory pathogens. Clin. Lab..

[18] Visseaux B., Collin G., Ichou H., Charpentier C., Bendhafer S., Dumitrescu M., Allal L., Cojocaru B., Desfrère L., Descamps D. (2017). Usefulness of multiplex PCR methods and respiratory viruses’ distribution in children below 15 years old according to age, seasons and clinical units in France: A 3 years retrospective study. PLoS ONE.

[19] Saarela E., Tapiainen T., Kauppila J., Pokka T., Uhari M., Kauma H., Renko M. (2019). Impact of multiplex respiratory virus testing on antimicrobial consumption in adults in acute care: A randomized clinical trial. Clin. Microbiol. Infect..

[20] Brendish N.J., Malachira A.K., Beard K.R., Ewings S., Clark T.W. (2018). Impact of turnaround time on outcome with point-of-care testing for respiratory viruses: A post hoc analysis from a randomised controlled trial. Eur. Respir. J..

[21] Doan Q.H., Kissoon N., Dobson S., Whitehouse S., Cochrane D., Schmidt B., Thomas E. (2009). A randomized, controlled trial of the impact of early and rapid diagnosis of viral infections in children brought to an emergency department with febrile respiratory tract illnesses. J. Pediatrics..

[22] Barlam T.F., Cosgrove S.E., Abbo L.M., Macdougall C., Schuetz A.N., Septimus E.J., Srinivasan A., Dellit T.H., Falck-Ytter Y.T., Fishman N.O. (2016). Implementing an antibiotic stewardship program: Guidelines by the Infectious Diseases Society of America and the Society for Healthcare Epidemiology of America. Clin. Infect. Dis..

[23] Kreitmeyr K., von Both U., Pecar A., Borde J.P., Mikolajczyk R., Huebner J. (2017). Pediatric antibiotic stewardship: Successful interventions to reduce broad-spectrum antibiotic use on general pediatric wards. Infection.

[24] Di Gennaro F., Marotta C., Amicone M., Bavaro D.F., Bernaudo F., Frisicale E.M., Kurotschka P.K., Mazzari A., Veronese N., Murri R. (2020). Italian young doctors’ knowledge, attitudes and practices on antibiotic use and resistance: A national cross-sectional survey. J. Glob. Antimicrob. Resist..

[25] Meskill S.D., O’Bryant S.C. (2020). Respiratory virus co-infection in acute respiratory infections in children. Curr. Infect. Dis. Rep..

[26] Shukla P.J., Behnam-Terneus M., Cunill-De Sautu B., Perez G.F. (2017). Antibiotic use by pediatric residents: Identifying opportunities and strategies for antimicrobial stewardship. Hosp. Pediatrics.

[27] Ko D.H., Kim H.S., Hyun J., Kim H.S., Kim J.S., Park K.U., Song W. (2017). Comparison of the Luminex xTAG Respiratory Viral Panel Fast v2 Assay With Anyplex II RV16 Detection Kit and AdvanSure RV Real-Time RT-PCR Assay for the Detection of Respiratory Viruses. Ann. Lab. Med..

